# Therapeutic drug monitoring-guided optimization of isavuconazole dose for ICU patients with refractory pulmonary aspergillosis: case report

**DOI:** 10.3389/fmed.2026.1693726

**Published:** 2026-03-16

**Authors:** Mingyue Yang, Xiaoshuang He, Qiuya Lu, Xiaolan Bian, Jie Fang, Yongjie Ding

**Affiliations:** 1 Department of Pharmacy, Ruijin Hospital, Shanghai Jiaotong University School of Medicine, Shanghai, China; 2 Department of Pharmacy, Yantai Yuhuangding Hospital, Yantai, China; 3 Department of Laboratory Medicine, Ruijin Hospital, Shanghai Jiaotong University School of Medicine, Shanghai, China; 4 Department of Pulmonary and Critical Care Medicine, Ruijin Hospital, Shanghai Jiao Tong University School of Medicine, Shanghai, China; 5 Institute of Respiratory Diseases, Shanghai Jiao Tong University School of Medicine, Shanghai, China; 6 Shanghai Key Laboratory of Emergency Prevention, Diagnosis and Treatment of Respiratory Infectious Diseases, Shanghai, China

**Keywords:** intensive care unit, isavuconazole, pharmacokinetics, pulmonary aspergillosis, therapeutic drug monitoring

## Abstract

Isavuconazole (ISA) is recommended as the first-line therapy for pulmonary aspergillosis. In intensive care unit (ICU) patients, ISA exhibits enhanced clearance and a larger volume of distribution but lower plasma concentrations, indicating the need for plausible dose adjustment. However, current evidence regarding off-label ISA dosing regimens for ICU patients is restricted to pharmacokinetic prediction modeling, with no published clinical studies available to date. In this case, a prolonged ISA-loading dosing regimen was applied to address previous standard ISA treatment failures; thereby, pharmacokinetic steady state was achieved with serial therapeutic drug monitoring (TDM)-guided dose optimization. This study illustrated a favorable clinical course associated with TDM-guided dosing adjustment, with no severe adverse reactions. Given the limited sample size, it is critical to investigate the clinical scenario further to adequately provide references for ISA dosing guidelines and TDM strategies for ICU practice.

## Introduction

Pulmonary aspergillosis (PA) caused by the environmental mold *Aspergillus fumigatus* constitutes a leading cause of global morbidity and mortality. As a ubiquitous opportunistic pathogen, *Aspergillus* primarily affects immunocompromised individuals, critically ill patients, and those with severe influenza (1). Among more than 300 species in the genus Aspergillus, *A. fumigatus* is the most prevalent environmental species and employs robust immune evasion strategies, such as molecular masking and immune modulation, to evade host defenses (2). The management of pulmonary aspergillosis is complicated by limited therapeutic options, drug interactions, adverse events, and drug resistance, resulting in elevated mortality rates (30–80%) (3, 4).

Isavuconazole (ISA) and voriconazole (VOR), second-generation triazole antifungals, are recommended as first-line therapies for PA (5). In contrast to VOR, ISA exhibits predictable pharmacokinetics (PK), lower interpatient variability, and a better safety profile (6). The standard ISA dosing regimen consists of a loading dose (LD) of 200 mg q8h for six doses, followed by a maintenance dose (MD) of 200 mg q24h. Initially, therapeutic drug monitoring (TDM) and dose adjustment for ISA were not recommended.

However, studies on ISA PK in critically ill patients have shown variable results (7–9). Hepatic dysfunction, hypoalbuminemia, increased volume of distribution (Vd), renal replacement therapy (CRRT), and extracorporeal membrane oxygenation (ECMO) may contribute to reduced plasma concentration of ISA (10–12). Meanwhile, limited studies have characterized the population pharmacokinetics (pPK) of ISA in ICU patients, with optimized dosing regimens proposed for this population through Monte Carlo simulations, as shown in Table 1. Nevertheless, real-world studies evaluating such off-label dosing strategies have not yet been conducted. In this study, we present the TDM of ISA in a case of refractory pulmonary aspergillosis requiring off-label dosing following standard-dose treatment failure. This report may provide evidence for the PK of high-dose ISA in ICU patients and illustrate the potential utility of TDM-guided dosing optimization in this challenging clinical context.

**Table 1:** Characteristics and results of published population pharmacokinetic models of isavuconazonium for ICU patients.

Study designs	Models	Data	Subject characteristics	Pharmacokinetic parameters	Covariates retain	PTA for a standard regimen	Dosage optimization	Ref.
Prospective study	NONMEM; allometrically scaled two-compartment model	152 samples from 29 ICU patients	RRT:7 (n) SOFA: 10 (3–18)	CL = 215 L/h V_1_ = 2,330 L Q = 2,590 L/h V_2_ = 28,200 L	Total body weight	35.8% reach the total AUC above 60 mg·h/L on day 14	When C_min_ at 24 h was below 1.0 mg/L, between 1.0 and 1.5 mg/L, or above 5.0 mg/L, daily maintenance doses were adjusted to 400 mg, 300 mg, and 100 mg, respectively.	(15)
Retrospective study	SAEM; one-compartment model with linear elimination	65 samples from 18 ICU patients with COVID-19-associated pulmonary aspergillosis	RRT: 6 (n) ECOM: 5 (n) SOFA: 5.5 (3.3–8.8)	CL = 3.97 L/h βCL RRT = 0.44 logL/h V_1_ = 837 L	RRT	19 and 6% at 72 h and 18 and 2% at 7 days for non-RRT and RRT patients, respectively. (>2 mg/L)	The regimen with a loading dose of 800 mg/day over 72 h and a maintenance dose of 400 mg achieved the target in 69 and 57% of cases at 72 h and 94 and 72% at 7 days for non-RRT and RRT patients, respectively.	(8)
Prospective, observational study	NONMEN; a one-compartment model with first-order elimination	71 samples from 24 ICU patients	RRT: 8 (n) ECOM: 7 (n) SAPS3: 63 (57–71.5)	CL = 5.36 L/h V_1_ = 122 L AUC = 37.31 mg·h/L	None	87.5% reached the optimal therapeutic target (>1 mg/L)	NA	(7)

PTA, probability of target attainment; CL, clearance; CV, coefficient of variation; V_1_, central volume of distribution; V_2_, peripheral volume of distribution; Q, intercompartmental clearance; AUC, area under the curve; RRT, renal replacement therapy; ECMO, extracorporeal membrane oxygenation.

## Case description

We present the case of a 55-year-old Chinese male patient (weight: 65 kg, height: 170 cm, body mass index (BMI): 22.49) who was admitted to Ruijin Hospital with chief complaints of pulmonary aspergillosis (PA), type II respiratory failure, type 2 diabetic ketoacidosis, and influenza A virus pneumonia. According to the Practice Guidelines for the Diagnosis and Management of Aspergillosis: 2016 Update by the Infectious Diseases Society of America, the patient met the diagnostic criteria for pulmonary aspergillosis. This diagnosis was supported by microbiological evidence—including a positive bronchoalveolar lavage fluid (BALF) culture, positive metagenomic next-generation sequencing (mNGS) for *A. fumigatus*, and an elevated BALF galactomannan index of 2.4 ng/mL, compatible CT imaging showing multiple infiltrates and areas of consolidation, and an underlying immunocompromising condition (type 2 diabetes). His history was significant for refractory pulmonary aspergillosis. Voriconazole (VOR) tablets and isavuconazole (ISA) sulfate tablets (an LD of 200 mg every 8 h for 48 h and an MD of 200 mg every 24 h), combined with caspofungin acetate injection and liposomal amphotericin B, were administered to treat aspergillosis without success at a local hospital during the month prior to presentation. The dose of liposomal amphotericin B was adjusted from 5 mg/kg to 3 mg/kg due to elevated creatinine levels prior to admission. Despite comprehensive treatment, the patient developed worsening hypoxemia and progression on computed tomography (CT), requiring emergent endotracheal intubation and mechanical ventilation, consistent with disease progression due to uncontrolled infection. The patient was subsequently admitted to our respiratory intensive care unit (RICU). Upon admission, the patient exhibited impaired consciousness and received mechanical ventilation through endotracheal intubation. Coarse breath sounds with bilateral moist rales were auscultated over both lung fields. Arterial blood gas analysis demonstrated a partial pressure of oxygen (PaO₂) of 112 mmHg and a partial pressure of carbon dioxide (PaCO₂) of 88 mmHg.

On admission, treatment with intravenous (IV) posaconazole (POS) and caspofungin, a regimen to which the patient had not been exposed, was initiated. However, the patient developed a fever of 38.7 °C, and a chest X-ray revealed increased bilateral lung markings. Given the failure of multiple prior standard antifungal therapies (voriconazole, posaconazole, isavuconazole, and amphotericin B), the multidisciplinary team decided to initiate an off-label regimen featuring an extended loading dose duration after thorough discussion and after obtaining informed consent from the patient’s family. On day 8, posaconazole was discontinued, and intravenous ISA was applied with an LD of 200 mg every 8 h for 72 h and an MD of 200 mg every 24 h. The trough concentration (C_min_) of ISA was strongly recommended. The patient’s hepatic and renal function, electrolyte profile, and cardiac function were closely monitored.

The blood sample was collected 30 min prior to ISA administration. All samples were extracted using the protein precipitation method. Calibration curves of isavuconazole were constructed by plotting the ratio of the peak areas of different concentrations of analyte to that of ISA with a weighting factor of 1/*x*^2^. The proposed calibration curves were linear, with linear regression coefficients above 0.90 in the range from 0.03 to 10.05 μg/mL. The intraday and interday precision and accuracy of the method were determined using the coefficient of variation (CV) values by analyzing six replicates of low quality control (LQC), medium quality control (MQC), and high quality control (HQC). The interday tests were completed in three batches over 2 days. The intraday and interday accuracy and precision results were between 1.77 and 4.63. The trough concentration (C_min_) of ISA tested is summarized in Table 2. On day 8, the patient’s serum potassium declined to 3.14 mmol/L but increased to 3.46 mmol/L with potassium chloride (KCl) supplementation.

**Table 2:** Medication regimen and ISA concentration.

Date	D8	D9	D10	D11	D12	D13	D16	D18	D19
Dose of ISA (mg/d)	600	600	600	200	200	200	200	200	200
C_min_ (mg/L)	/	5.64	6.13	7.51	6.66	4.63	2.30	1.73	1.78

Following treatment, the patient remained afebrile with a peak temperature of 37.2 °C. The targeted next-generation sequencing (tNGS) analysis of BALF showed a marked reduction in *A. fumigatus* sequence reads from 1,144 to 287, accompanied by a negative G-test. Bedside chest radiography showed significant resolution of bilateral pulmonary infiltrates and consolidation. However, it is important to note that these improvements occurred in the context of multiple concomitant therapies, including continued caspofungin therapy and comprehensive supportive care (mechanical ventilation optimization, glycemic control, and nutritional support), which precludes the definitive attribution of clinical improvement solely to ISA dose optimization. The fungal infection was effectively controlled under the current regimen. However, considering the drug’s unavailability and the associated economic burden for the patient, therapy was switched to VOR on day 21. By day 21, the vital signs of the patient had stabilized. Given the drug availability and affordability, ISA was discontinued, and IV VOR was initiated at a dose of 200 mg every 24 h. The TDM of VOR was routinely conducted, with the results shown in Table 3. The (1–3)-β-D-glucan level was 13.33 pg/mL on day 22, whereas it had been undetectable in previous assays. Hepatic safety monitoring showed preserved liver function, with satisfactory levels of alanine aminotransferase (ALT), aspartate aminotransferase (AST), total bilirubin, and direct bilirubin.

**Table 3:** Medication regimen and VOR concentration.

Date	D25	D27	D30
Dose of VOR (mg/d)	400	400	400
C_min_ (mg/L)	0.2	0.1	0.2

The pharmacogenetic testing of the patient’s enzymes was performed on day 18 and revealed CYP2C19*1/*1 (extensive metabolizer), CYP3A4*1/*1 (extensive metabolizer), CYP3A5*1/*3 (intermediate metabolizer), and CYP2D6 *1/*10 (extensive metabolizer).

**Figure 1:**
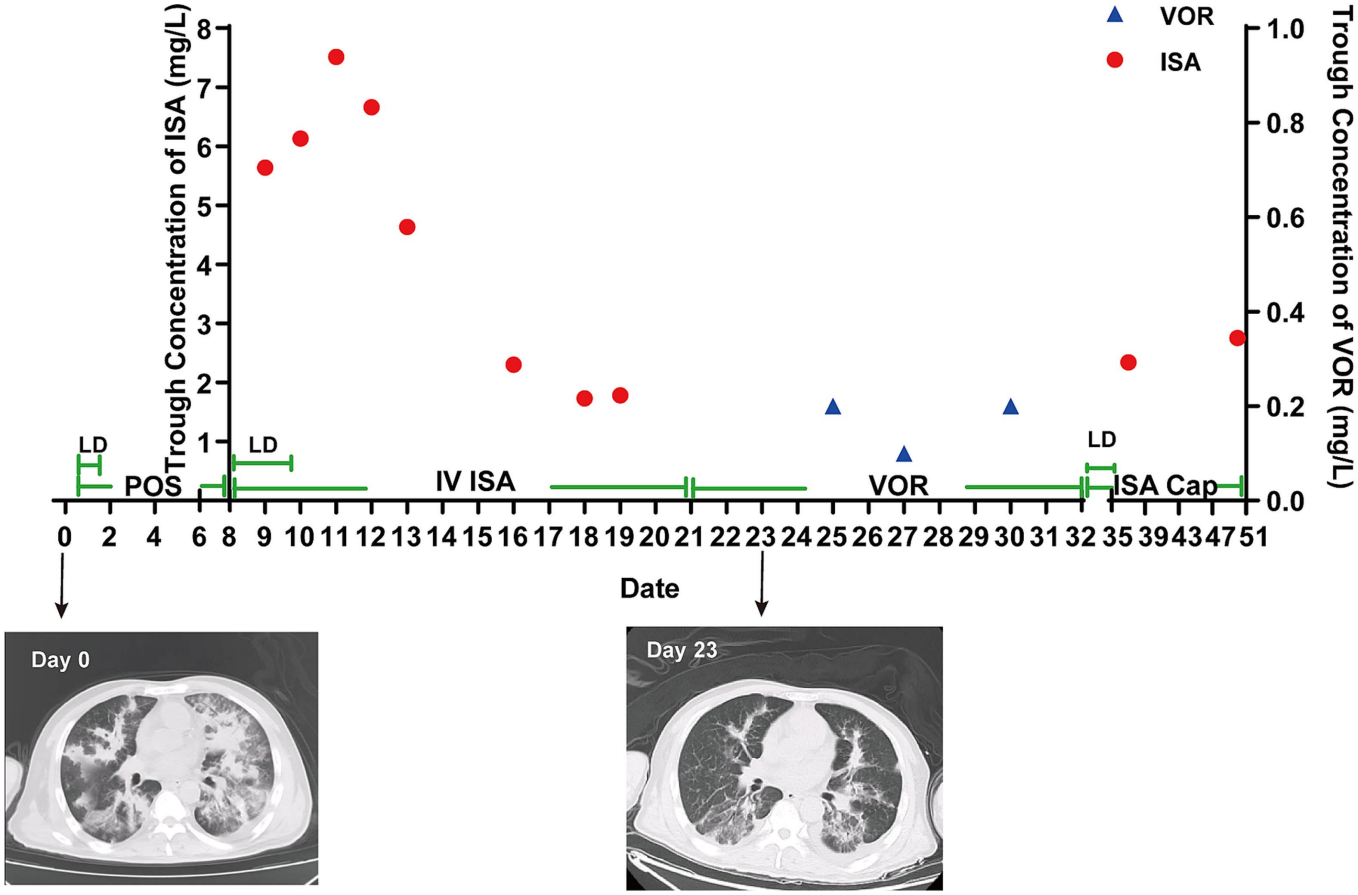
Clinical course and trough concentration of ISA and VOR.

On day 33, VOR was replaced with ISA capsules (Cap). The patient was administered an LD of 200 mg every 8 h for 72 h, followed by an MD of 200 mg every 24 h. This regimen was supported by subtherapeutic plasma concentrations of VOR, increased G-test levels, and the onset of chest tightness and dyspnea in the patient. On day 37, the C_min_ of ISA was 2.34 mg/L. The patient exhibited a progressive decline in ventilator dependence, undergoing weaning and eventual extubation on day 38. On day 43, the patient exhibited stable vital signs and achieved ambulation capacity after rehabilitation training. Thereafter, the patient was transferred to the respiratory department for further management and, on day 50, the C_min_ of ISA was 2.75 mg/L, suggesting recovery. The patient was discharged on day 51. The time course of ISA concentrations throughout the treatment is shown in Figure 1.

## Discussion

Invasive fungal infections account for 16% of all ICU-acquired infections and are associated with an in-hospital mortality rate of 32.4% (13). In this case of refractory pulmonary aspergillosis, a favorable clinical course was observed with a synergistic regimen of ISA dose escalation (LD: 200 mg q8h for 72 h; MD: 200 mg qd) and caspofungin (70 mg qd). However, given the multifactorial nature of the patient’s management—including the sequential use of multiple antifungal agents, concomitant caspofungin therapy, and comprehensive critical care support—the clinical outcome should not be attributed solely to the ISA dosing strategy. Nonetheless, this case illustrates the feasibility and potential utility of TDM-guided dose optimization in achieving target drug exposures in a complex clinical setting.

Current evidence is insufficient to establish a valid pharmacodynamic target for ISA. Variability in target concentrations is observed across studies, with 1 mg/L or 2 mg/L proposed as the efficacy threshold of ISA in a number of reports (7, 8). The trough concentrations predominantly fell within the range of 1–7 mg/L, with no concentration-dependent relationship observed in the isavuconazole versus voriconazole for primary treatment of invasive mould disease caused by Aspergillus and other filamentous fungi (SECURE) (12). Furthermore, real-world studies have suggested that a high level of C_min_ (>5 mg/L) is associated with an increased risk of adverse effects (13).

ICU populations show highly variable PK, driven by organ dysfunction, capillary leakage, and fluid redistribution. Low ISA exposure in ICU patients has been observed in several studies, challenging optimal clinical efficiency. Accumulating evidence from pharmacokinetic studies has revealed significantly reduced ISA exposure in critically ill patients, raising concerns about overdosing to attain therapeutic targets in this population. Cerezuela et al. documented a median C_min_ of 1.76 mg/L (IQR: 1.02 mg/L) in critically ill patients (7). Similarly, another pharmacokinetic study of ISA in ICU patients reported a median C_min_ of 1.87 mg/L [1.29–2.25 mg/L] (8). Notably, these values were substantially lower than those observed in real-world studies (mean: 2.98 ± 1.91 mg/L) and clinical trials (mean: 3.30 ± 2.18 mg/L) (6, 14). Due to the limited research on pharmacokinetics of ISA in such special populations, the optimal dosing regimen for these individuals remains uncertain. Based on the Monte Carlo simulations, the regimen with an LD of 800 mg/day for 72 h contributes to an improved probability of target attainment (PTA) in ICU patients at both 72 h and 7 days (8). Another study reported obtaining the first pharmacokinetic baseline value 24 h after drug initiation to guide early dose optimization through TDM (15).

Under the standard dosing strategy, TDM performed after completion of the loading dose phase (day 3) is considered the first reliable indicator of steady-state conditions (16). Despite adherence to the prolonged LD regimen and the C_min_ peak post-LD completion in this patient, a progressive decline was observed through day 6 after administration without achieving steady state. In contrast, model-predicted concentrations have been reported to demonstrate an overall ascending trend, with higher PTA at day 7 vs. 72 h (34% vs. 41%) (8). This pharmacokinetic discrepancy suggests potential limitations of current prediction models, and idiosyncrasy cannot be excluded. In light of this case, TDM is warranted on days 4 and 7 post-initiation for patients receiving a 72-hour loading dose, to ensure optimal exposure and minimize toxicity.

Unanticipated VOR pharmacokinetic variability persisted in this case. The TDM of VOR ranged between 0.1 and 0.2 mg/L, substantially below the established therapeutic window of 1–5.5 mg/L (17). VOR metabolism is catalyzed by CYP2C19, CYP3A4, and CYP2C9 in the liver, whereas CYP3A4 and CYP3A5 play vital roles in ISA metabolism. Consequently, hepatic function, drug–drug interactions, and genetic polymorphisms have been identified as factors affecting the PK of both VOR and ISA. The patient presented with neither obesity nor severe hepatic dysfunction. The patient concurrently received the moderate CYP450 inhibitor omeprazole (40 mg daily) and the enzyme inducer prednisone (10 mg daily), which may exert modest effects on VOR concentrations without requiring dose adjustment (18). The effect of moderate and weak inhibitors of CYP3A4/5 on the PK of ISA was investigated, and none of them were finally included as covariates (18, 19). The patient’s genotype was identified as CYP2C19*1/*1, CYP3A4*1/*1, and CYP3A5*1/*3, a profile commonly observed in Asian populations (20). For patients with CYP2C19*1/*1, dose adjustment is not typically recommended (21). Currently, no clinical studies have shown a clinically significant influence of these genotypes on the PK of VOR and ISA (22, 23). Collectively, existing pharmacogenomic evidence is insufficient to demonstrate the presence of clinically significant genetic variants affecting the PK of ISA in this patient.

Safety represents a critical focus in clinical medication. Hypokalemia was identified in this case when the C_min_ of ISA was 5.64 mg/L; however, hypokalemia persisted for a week after the completion of ISA administration. In addition, this patient received concomitant therapy with methylprednisolone and caspofungin, both of which have documented hypokalemic potential in clinical practice. According to the World Health Organization–Uppsala Monitoring Centre’s (WHO-UMC) causality assessment system, the association between hypokalemia and ISA in this patient was categorized as “unlikely.” Meanwhile, hypokalemia was successfully managed with KCl supplementation without notable clinical consequences.

In conclusion, this case report demonstrates the implementation of individualized ISA therapy guided by TDM in an ICU patient, suggesting that TDM may be necessary to adjust ISA dosing strategies in ICU patients. Previous studies have suggested that ISA may exhibit suboptimal plasma concentrations in ICU populations. In this case, the patient initially failed standard-dose ISA therapy. Subsequently, an extended LD regimen was implemented with intensive TDM. The monitoring revealed that peak C_min_ was achieved immediately after completion of the LD, while steady-state plasma concentration was reached 7 days post-initiation. However, as a single case report, these findings require validation through large-scale clinical trials to establish optimal dosing strategies and TDM timing for ISA in critically ill patients.

## Data Availability

The original contributions presented in the study are included in the article/supplementary material, further inquiries can be directed to the corresponding authors.
